# Isolation and Characterization of *Lactobacillus* spp. from Kefir Samples in Malaysia

**DOI:** 10.3390/molecules24142606

**Published:** 2019-07-17

**Authors:** Noorshafadzilah Talib, Nurul Elyani Mohamad, Swee Keong Yeap, Yazmin Hussin, Muhammad Nazirul Mubin Aziz, Mas Jaffri Masarudin, Shaiful Adzni Sharifuddin, Yew Woh Hui, Chai Ling Ho, Noorjahan Banu Alitheen

**Affiliations:** 1 Department of Cell and Molecular Biology, Faculty of Biotechnology and Biomolecular Sciences, Universiti Putra Malaysia, UPM, Serdang, Selangor 43400, Malaysia; 2 China-ASEAN College of Marine Sciences, Xiamen University Malaysia, Sepang, Selangor 43900, Malaysia; 3 Department of Bioprocess Biotechnology, Malaysian Agriculture Research Development Institute, Serdang, Selangor 43400, Malaysia

**Keywords:** lactic acid bacteria, *Lactobacillus*, kefir, kefir drink, probiotics

## Abstract

Kefir is a homemade, natural fermented product comprised of a probiotic bacteria and yeast complex. Kefir consumption has been associated with many advantageous properties to general health, including as an antioxidative, anti-obesity, anti-inflammatory, anti-microbial, and anti-tumor moiety. This beverage is commonly found and consumed by people in the United States of America, China, France, Brazil, and Japan. Recently, the consumption of kefir has been popularized in other countries including Malaysia. The microflora in kefir from different countries differs due to variations in culture conditions and the starter media. Thus, this study was aimed at isolating and characterizing the lactic acid bacteria that are predominant in Malaysian kefir grains via macroscopic examination and 16S ribosomal RNA gene sequencing. The results revealed that the Malaysian kefir grains are dominated by three different strains of *Lactobacillus* strains, which are *Lactobacillus harbinensis*, *Lactobacillusparacasei*, and *Lactobacillus plantarum*. The probiotic properties of these strains, such as acid and bile salt tolerances, adherence ability to the intestinal mucosa, antibiotic resistance, and hemolytic test, were subsequently conducted and extensively studied. The isolated *Lactobacillus* spp. from kefir H maintained its survival rate within 3 h of incubation at pH 3 and pH 4 at 98.0 ± 3.3% and 96.1 ± 1.7% of bacteria growth and exhibited the highest survival at bile salt condition at 0.3% and 0.5%. The same isolate also showed high adherence ability to intestinal cells at 96.3 ± 0.01%, has antibiotic resistance towards ampicillin, penicillin, and tetracycline, and showed no hemolytic activity. In addition, the results of antioxidant activity tests demonstrated that isolated *Lactobacillus* spp. from kefir G possessed high antioxidant activities for total phenolic content (TPC), total flavonoid content (TFC), ferric reducing ability of plasma (FRAP), and 1,1-diphenyl-2-picryl-hydrazine (DPPH) assay compared to other isolates. From these data, all *Lactobacillus* spp. isolated from Malaysian kefir serve as promising candidates for probiotics foods and beverage since they exhibit potential probiotic properties and antioxidant activities.

## 1. Introduction

Fermented foods and beverages have been staples for human diet for thousands of years [1]. Fermentation plays a major role in food preservation and nutritional contents through the enrichment of substrates such as vitamins, proteins, essential fatty acids, and amino acids [2]. The examples of fermented foods that are widely known include wine, beer, yogurt, kimchi, milk, cereals, soybeans, fruits, and fish [3]. The unique flavor and texture of the fermented foods are contributed to by the presence of microorganisms and their byproduct produced during fermentation. These microbes are referred to as “probiotics” [4].

The study of probiotic products is one of the fastest growing ventures among other functional foods due to an increase in consumer awareness towards its multitude of beneficial effects on health [5]. Probiotics are defined as living microorganisms that offer health benefits to the host when consumed in an adequate quantity [6]. Probiotics are functional foods that have been demonstrated to be effective treatments to control several diseases such as inflammatory bowel disease, allergy, diarrhea, etc. [7]. The gut is the key target for probiotics foods because it acts as the main interface between diet and metabolic pathways in human health as it modulates the intestinal microflora [8]. Many genera and species of microorganisms can be considered as potential probiotics, but the genera that are commercially used in probiotic foods are the *Lactobacillus* and *Bifidobacterium* [9]. *Lactobacillus* acts as an important probiotic because of its strain-specific properties, which are beneficial to health, mainly towards the microflora of the gastrointestinal tract [10]. It also has been declared to have generally regarded as safe (GRAS) status and is regulated by the FDA (food and drug administration) for human and animal consumption [11].

Kefir is one of the fermented drinks that has been consumed since long ago; it is an acidic and viscous drink, possesses a sour taste, is slightly alcoholic, and can be easily digested [12]. The kefir beverage is produced through the fermentation of kefir grains containing yeasts, lactic, and acetic acid bacteria embedded in its exopolysaccharide matrix [13]. However, *Lactobacillus* makes up the major microbial population found in all kefir grains, implying the importance of this group of bacteria in the production of the kefir beverage [14]. The microflora composition and predominant bacteria in kefir may vary depending on the kefir origin, due to the substrate used in the fermentation process and culture maintenance method [15]. Based on previous studies, kefir is reported to have good benefits to our health such as stimulation of immune system, anti-obesity, and can relieve inflammatory bowel diseases [16,17,18]. Recent studies have reported the uses of kefir in terms of food applications, cosmetic purposes, and industries due to the physicochemical, microbiological, and bioactive compound contained in kefir [19,20,21]. Kefir has been used as an ingredient in wine production, kefir cereal-based beverages, kefir facial mask, and dyes [22]. Further studies into the characterization of specific microbial compositions in kefir grains, mainly the *Lactobacillus* species, is of utmost interest, especially from Malaysia where the production conditions vary between regions. This knowledge will facilitate the development of desirable starter cultures that can be further used to produce kefir products according to the industrial standard. Thus, this study is expected to contribute to the knowledge on the composition of predominant *Lactobacillus* in Malaysia’s kefir grains and their benefits to consumers, as no scientific data are available about its probiotic potential. This study aims to isolate and identify predominant *Lactobacillus* strains from 10 different sources of Malaysia’s kefir samples. The resulting predominant *Lactobacillus* strains for each kefir sample were studied for their antioxidant activities and its probiotic potential.

## 2. Results

### 2.1. Isolation and Identification of Lactobacillus spp. from Kefir

**Figure 1:**
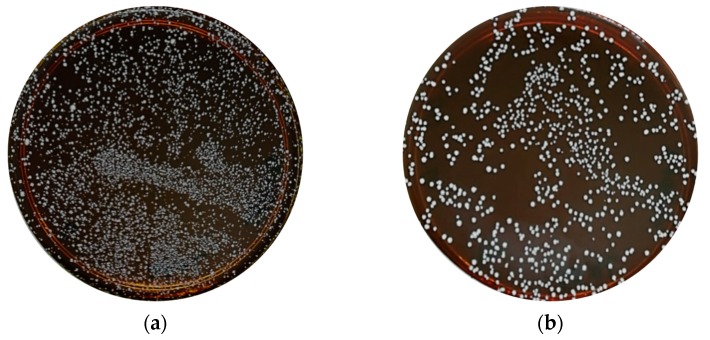
Representative figures of the morphological analysis of the isolated *Lactobacillus* spp. from kefir sample on de Man, Rogosa and Sharpe (MRS) agar medium. (**a**) Isolated *Lactobacillus* colonies from kefir sample on MRS media; (**b**) single-screened isolated *Lactobacillus* from kefir sample on MRS media.

**Figure 2:**
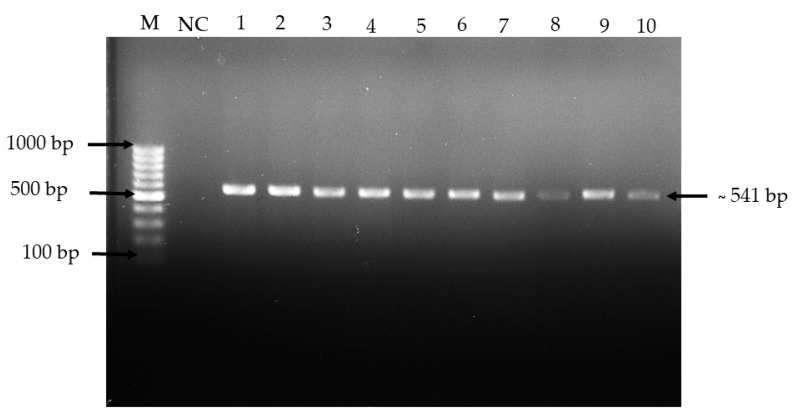
Agarose gel electrophoresis of PCR products of 16S rRNA gene from *Lactobacillus* spp. isolated from 10 kefir samples. M; DNA molecular marker-100 bp; NC: Negative control; Lane 1–10: Isolated *Lactobacillus* spp. from 10 kefir samples A–J, respectively.

The bacterial isolates were examined based on culture characteristics and macroscopic analysis, as shown in Figure 1. It was observed that most of the isolates formed off-white pinhead colonies typical of *Lactobacillus* spp. The identification of isolated *Lactobacillus* spp. from kefir samples was determined using 16S rRNA gene by PCR analysis and yielded a single amplicon band at approximately 541 bp, as shown in Figure 2. The sequences were aligned to the query sequences of the GenBank 16S rRNA sequences database, resulting in identities of known sequences of 99–96%, as shown in Table 1 [23,24]. The three isolated *Lactobacillus* spp. most probably predominant from the kefir samples were *L. harbinensis, L. paracasei, and L. plantarum.*

### 2.2. Tolerance to Low pH Condition of the Isolates

**Figure 3:**
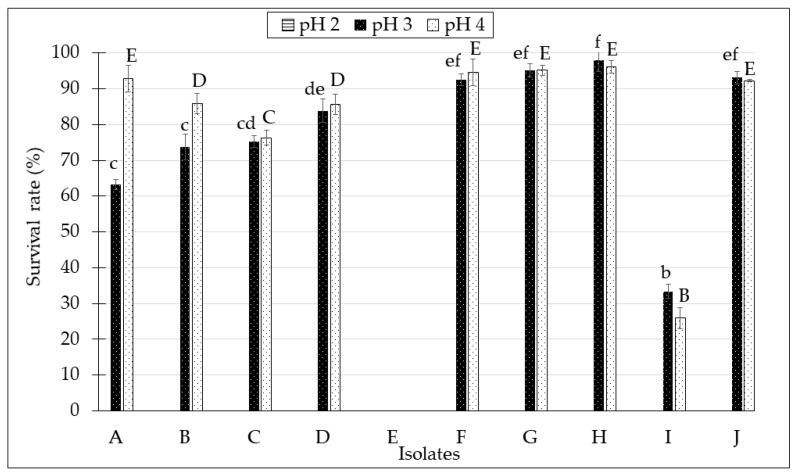
Survival rate of isolated *Lactobacillus* spp. from kefir samples under acidic conditions (pH 2.0, pH 3.0, and pH 4.0) for 3 h. All data were expressed as mean ± SD. ^a–f^ Different lowercase letter in superscript on the bar graph indicates significant different (*p* < 0.05) for pH 3. ^B–E^ Different uppercase letter in superscript on the bar graph indicates significant different (*p* < 0.05) for pH 4.

The effects of simulated gastric juice on the survival rate of the isolated *Lactobacillus* spp. from 10 kefir samples at incubation time for 3 h is shown in Figure 3. In this study, the isolates were subjected for different pH tolerance (pH 2.0, pH 3.0, and pH 4.0). From the data, isolated *Lactobacillus* spp. from kefir H showed a remarkable surviving rate of 98.0 ± 3.3% and 96.1 ± 1.7% after exposure to pH 3.0 and pH 4.0, respectively.

However, *Lactobacillus* spp. isolated from kefir E was unable to survive in all pH condition (pH 2, 3, and 4). All isolates tested did not survive pH 2.0, suggesting that the samples cannot withstand the most acidic condition of gastric juice. However, most of the isolated *Lactobacillus* spp. from kefir samples was able to tolerate the moderate pH levels of pH 3.0 and pH 4.0.

### 2.3. Bile Salt Tolerance Test

**Figure 4:**
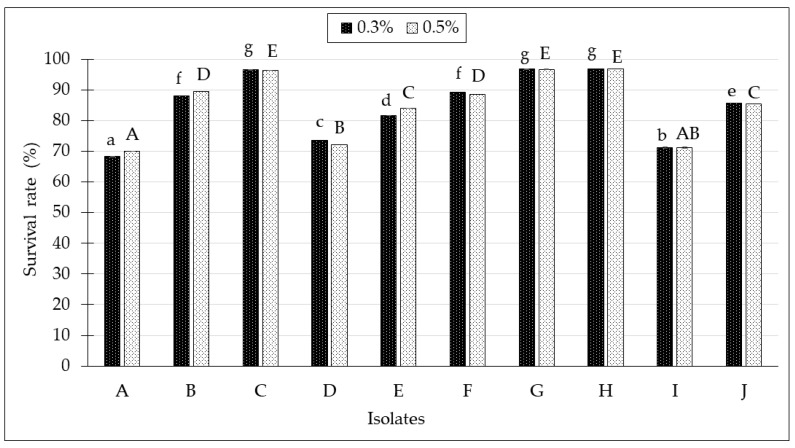
Survival rates of isolated *Lactobacillus* spp. from kefir samples in 0.3% and 0.5% bile salt conditions for 3 h. All data were expressed as mean ± SD. ^a–g^ Different lowercase letter in superscript on the bar graph indicates significant different (*p* < 0.05) for 0.3% bile salt concentration. ^A–G^ Different uppercase letter in superscript on the bar graph indicates significant different (*p* < 0.05) for 0.5% bile salt concentration.

All *Lactobacillus* isolates were subjected to different bile concentrations, 0.3% and 0.5%, at an incubation time of 3 h. As shown in Figure 4, all the isolated *Lactobacillus* spp. from kefir samples survived at 0.3–0.5% bile concentration after incubation. The *Lactobacillus* spp. from kefir H conferred the highest survival rate at 0.3% and 0.5% bile concentration, with a survival rate of 96.89 ± 0.02% and 96.84 ± 0.02%, respectively. Subsequently, the survival rate in bile salt condition was followed by isolated *Lactobacillus* spp. from kefir G and kefir C.

### 2.4. Adherence Assay

**Figure 5:**
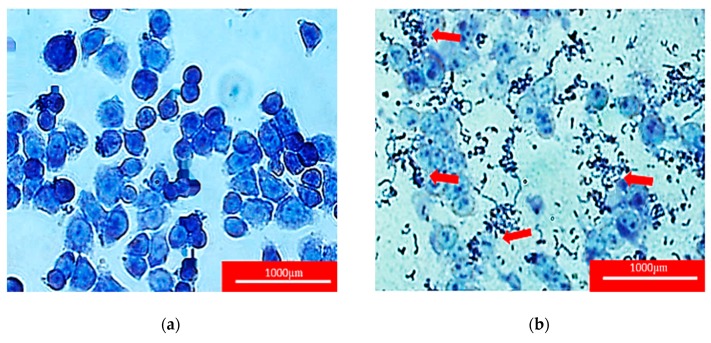
The red arrows indicate the adhesion of isolated *Lactobacillus* spp. from kefir samples to HT-29 cell line under bright field microscope 400× magnification after staining with Giemsa stain. (**a**) Healthy HT-29 cell line without any treatment; (**b**) representative figure of adhesion isolated *Lactobacillus* from kefir A–J to HT-29 cell line.

**Figure 6:**
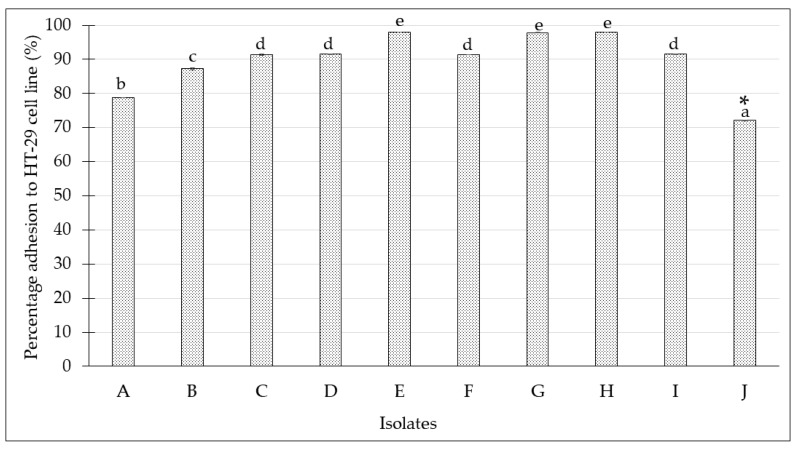
Percentage of adhered isolated *Lactobacillus* spp. from kefir samples to HT-29 cell line. All data were expressed as mean ± SD. Values not sharing a common superscript are significantly different (*p* < 0.05). * indicates mean values (*p* < 0.05).

The adherence ability of the isolated *Lactobacillus* spp. from kefir samples on HT-29 cell line was determined by direct microscopic examination using Giemsa staining. The results indicated that all the isolated *Lactobacillus* spp. from kefir samples were categorized as strongly adhesive due to the observation of adherence for more than 100 bacteria on the cells, as indicated by the arrow, as shown in Figure 5b. The number of bacteria adhered to the cells was determined by colony count on MRS agar, which was collected after trypsinization as it enables a total enumeration of the bacteria attached to HT-29 cell line. The adherence ability was expressed as percentage of adhered isolates to the number of isolates added, as depicted in Figure 6. The highest level of adherence was observed in the isolated *Lactobacillus* spp. from kefir H with percentage of adherence ability at 96.3 ± 0.01%. In addition, the adhesion ability of isolated *Lactobacillus* spp. from kefir J was significantly lower (*p* < 0.05) than other isolates.

### 2.5. Scanning Electron Microscopy of Adhered Isolated Lactobacillus spp. toward HT-29 Cells

Only one sample with the highest adhesion ability from the previous adherence assay was chosen for further analysis by viewing under scanning electron microscope.

**Figure 7:**
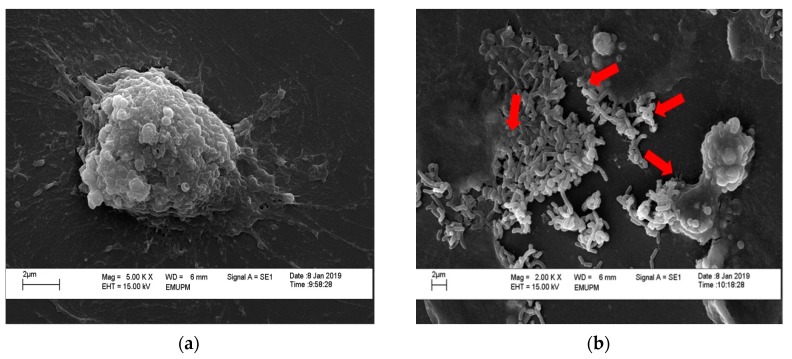
Scanning electron microscope (SEM) analysis of HT-29 cells where the *Lactobacillus* spp. adheres to the surface monolayer of cells. (**a**) Healthy HT-29 cells without any treatment; (**b**) *Lactobacillus* spp. from kefir H. Red arrow indicates attachment of isolated *Lactobacillus* spp. from kefir H to HT-29 cells.

This was done in order to obtain a greater insight into the morphology of the isolated *Lactobacillus* spp. from kefir that adhered onto HT-29 cells. Both untreated and bacterial-treated HT-29 cells were observed under scanning electron microscope, as shown in Figure 7. Figure 7a showed healthy HT-29 cells under 5000× magnification to get a better view of healthy cells without treatment of isolated *Lactobacillus* spp. from kefir. Figure 7b showed the presence of rod-shaped *Lactobacillus* forming chains and adhering on the surface of HT-29 cell under 2000× magnification.

### 2.6. Antibiotic Susceptibility Test

**Figure 8:**
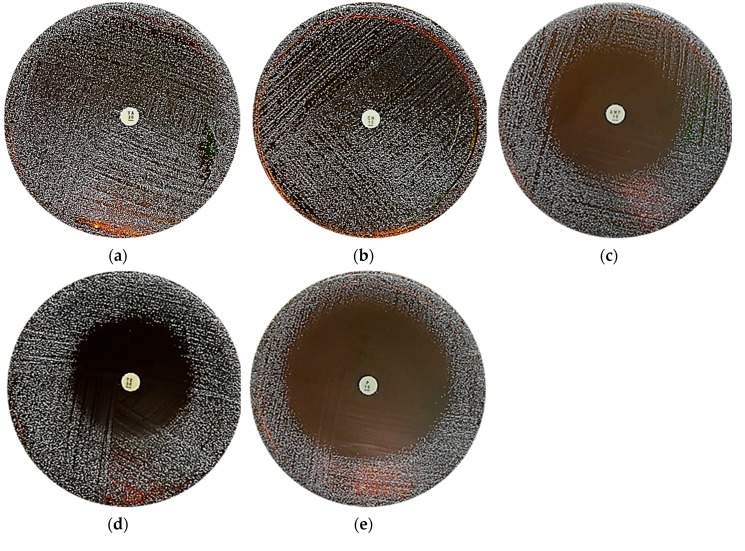
Antibiotic susceptibility test using disk diffusion method on MRS agar, where (**a**) vancomycin (VA) and (**b**) gentamicin (CN) antibiotics showed no zone of inhibition while (**c**) ampicillin (AMP), (**d**) tetracycline (TE), and (**e**) penicillin (P) antibiotics contain zones of inhibition.

All isolates were analyzed for their tolerance towards antibiotics due to safety considerations towards the threat of antibiotic resistance in bacteria. The antibiotic susceptibility test was done by disc diffusion method by measuring the zone of inhibition towards vancomycin (VA), gentamicin (CN), ampicillin (AMP), tetracycline (TE), and penicillin (P), as shown in Figure 8. The results for this assay are as depicted in Table 2 and expressed as resistant (−), moderately susceptible (+), susceptible (++), and very susceptible (+++). Most of the isolates were found to be resistant towards gentamicin and vancomycin, and very susceptible towards ampicillin, tetracycline, and penicillin antibiotics with inhibition zones ranging between 31–55 mm.

### 2.7. Hemolytic Test

All isolates did not present any hemolysis on the agar blood plates. All isolates were γ-hemolytic.

### 2.8. Antioxidant Assay

The antioxidant activities of all isolates were evaluated using the supernatant of the isolates, after incubation for 48 h at 37 °C by measuring the total phenolic content (TPC), total flavonoid content (TFC), ferric reducing ability of plasma (FRAP), and radical scavenging activity 1,1-diphenyl-2-picryl-hydrazine (DPPH). Table 3 depicts the antioxidant activities of all samples. Based on the overall results for all assays, the isolated *Lactobacillus* spp. from kefir G exhibited higher antioxidant activities as compared to other isolates based on the tested antioxidant assays. Among the 10 isolates, isolated *Lactobacillus* spp. from kefir G showed to have significantly high total phenolic content with 115.97 ± 7.22 mg gallic acid equivalent (GAE)/mg protein, followed by isolated *Lactobacillus* spp. from kefir H with 99.20 ± 4.46 mg GAE/mg protein. The same trend was observed for the TFC, FRAP, and DPPH assays, where the highest antioxidant activity was observed in isolated *Lactobacillus* spp. from kefir G, followed by isolate H. On the other end, isolated *Lactobacillus* spp. from kefir A had significantly low FRAP activity, while isolates from kefir D, I, and J had the lowest radical scavenging activity according to the DPPH assay.

## 3. Discussion

Kefir, which is made up of complex microbiota mainly *Lactobacillus* spp., has been suggested as a potential reservoir for probiotics [23]. Benefits of probiotics to human health includes aiding in balancing gut microflora by increasing number of microbes in the diet, triggering the immune system to combat pathogens, and antimicrobial properties [25,26]. In order to be classified as such, the *Lactobacillus* strain needs to fulfil certain criteria of probiotics such as being able to survive under extreme conditions (low pH, presence of bile salts), able to adhere to intestinal cells, and being non-pathogenic, in order to successfully colonize in the human and animal gastrointestinal tract [26]. In this study, results from an isolation study revealed that the 10 kefir samples were most probably predominated by three different *Lactobacillus* strains, namely *L. plantarum*, *L. harbinensis*, and *L.paracasei*. These results showed that different microflora strains were present in kefir grains, possibly influenced by various factors such as the origin of the kefir grains, the type of the substrate, fermentation condition, and culture maintenance method [15,27]. Similar results had been previously reported by Garofalo et al. [28] whereby the microflora species in Italian kefir grains were influenced by culturing method.

The first criterion evaluated in this study was the ability of the probiotic bacteria in kefir to tolerate low pH conditions, as it potentially indicates the ability of the bacterial strains to withstand the gastric juice in human stomach [29]. The lowest pH recorded in the human stomach is around pH 1.5, which normally occurs when a person is fasting. Good probiotic strains should be able to thrive in growth conditions of at least at pH 3.0 in the stomach, considering the influence of food matrix buffering capacity. Therefore, in previous studies, tolerance towards pH 3.0 has been tested in most of the in vitro assays [29,30,31,32]. The 3 h incubation time is necessary as the maximum incubation time to reflect the time that food lasts in the stomach before being digested [33]. From the results, none of the isolates survived the lowest pH (pH 2), which may be due to the extreme acidic condition. Other studies also confirmed that exposing the bacterial strains to gastric acid with pH ≤ 2 caused an intensive reduction in the viability count of the bacteria [34,35]. In this study, the isolated *Lactobacillus* spp. from kefir A, B, C, D, F, G, H, I, and J were able to survive at pH 3.0 and pH 4.0. However, there are inconsistencies in their survival rates, which might be due to their varying adaptation abilities towards acid at the time of their presence in MRS broth. These data were very similar to the study conducted by Tokatlı et al. [36], where they found that different isolated *Lactobacillus* spp. from pickles had varying survival rates in acidic conditions, which are due to the adaptation of the strains that have strain-specific properties.

The probiotics should also be able to withstand the bile acid concentration in the liver, which is synthesized from cholesterol and secreted from the gall bladder into the duodenum [37]. The ability of the isolates to survive in bile salts helps with the colonization and metabolic activity of bacteria in the small intestine of the host [38]. The bile concentration in human ranges between 0.3% to 0.5%, but some studies have suggested that the bile concentration varies depending on the diet composition and secretion of pancreatic enzymes [39,40]. In this bile salt tolerance test, the results showed that all *Lactobacillus* spp. isolated from kefir were tolerant towards 0.3% and 0.5% bile salt concentration. These findings are in accordance with a previous study by Mahmoudi et al. [41], where *Lactobacillus* spp. isolated from sheep and goat milk could withstand the bile salt concentrations of 0.3% and 0.5%.

In addition, probiotic strains must be able to adhere to the intestinal mucus in order to be colonized and established in the intestine [42]. In this study, all bacteria indicated excellent abilities to adhere and colonize on the intestinal cells (Figure 5b and Figure 6). The higher adherence ability of isolated *Lactobacillus* spp. from kefir H might be due to the strain’s higher ability to adhere to mucin and the epithelial cell culture. These data were similar to previous studies, which stated that the adherence ability is strain specific due to the difference in the receptors for bacterial adhesions on mucus and may also be influenced by the different origin of the kefir grains of which they were isolated from [43,44]. Based on the scanning electron microscopy results (Figure 7b), it was depicted that our isolated *Lactobacillus* spp. from kefir could also be regarded to have excellent adhesion property, contributing positively to its potential as probiotics. The isolated *Lactobacillus* spp. from kefir H had strong binding affinity, adhered to the HT-29 cells, and auto-aggregated to each other. The data obtained in this study are supported by Muryany et al. [45], who showed the auto-aggregative pattern of bacterial attachment on HT-29 cells of isolated *Lactobacillus* spp. from Malaysian fermented fish (Pekasam).

Probiotic bacteria have the ability to harbor intrinsic and mobile genetic elements that confer resistance to a wide variety of antibiotics. High amounts of probiotics in dietary supplements can establish a reservoir of antibiotic-resistant genes in the human gut and transfer to pathogens that share the same intestinal habitat, which is dangerous and needs to be prevented [46]. Therefore, it is necessary to determine the antibiotic resistance of the isolated *Lactobacillus* spp. to avoid serious clinical threats. In this study, it was observed that all isolates, A to J, were very susceptible to ampicillin, penicillin, and tetracycline. The obtained results were in accordance with the findings by Georgiva et al. [47], which showed that *Lactobacillus* are generally sensitive to beta-lactams antibiotics, such as ampicillin and penicillin, and broad-spectrum antibiotics like tetracycline. On the other hand, all isolates tested were resistant towards vancomycin and gentamycin. The antibiotic resistance of the isolates is non-transferable between isolates and species due to the fact that *Lactobacillus* species are intrinsically resistant to vancomycin and gentamycin. This is mainly attributed to two factors; the presence of d-alanine—d-alanine ligase-related enzymes prevent vancomycin from binding at the cytoplasmic end of their cell walls—and the absence of cytochrome-mediated electron transport, which mediates drug uptake [36]. Lastly, hemolysis is another well-known virulence factor among pathogenic microorganisms. All isolates were tested for hemolytic activity. All isolated *Lactobacillus* spp. from kefir were γ-hemolytic, which indicated no hemolysis on blood agar plates. This finding was similar to a previous study that revealed *Lactobacillus* spp. possess no hemolytic activity [48].

**Figure 9:**
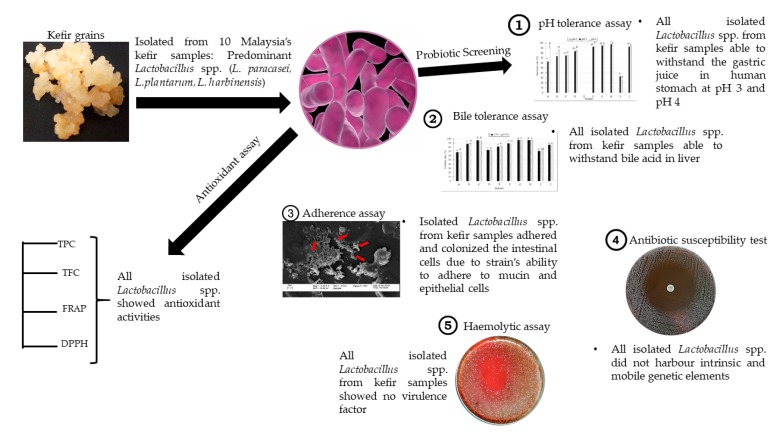
Summary of isolation and characterization of *Lactobacillus* spp. from kefir samples in Malaysia involving probiotic screening and antioxidant assays.

*Lactobacillus* spp. are used as alternative natural antioxidants that can prevent damage due to oxidative stress of free radicals in the host [24]. *Lactobacillus* spp. are capable of metabolizing phenolic and flavonoids compounds as their end product during fermentation [49,50]. The ability to metabolize the compounds is strain- or species-specific [51]. The increase of phenolic and flavonoids compounds during enzymatic hydrolysis of the lactic acid bacteria during fermentation lead to the increase of the antioxidant activities [51,52]. The results of this study showed that all isolated *Lactobacillus* spp. from kefir had different abilities to metabolize phenolic and flavonoids compounds, which in turn, contributed to their different antioxidant activities (Table 3). During microbial fermentation, there was an increase in acidic condition, which liberated bound flavonoid and phenolic components, making them more bioavailable, which in turn, reflected in the increase of flavonoid content in the TFC assay and the increase in phenolic content in the TPC assay [53]. These data were in agreement with Xiao et al. [54], who found that higher total phenolic content in fermented soy whey may result in the increase of total flavonoid content during *L. plantarum* fermentation. The ferric reducing ability of plasma (FRAP) and 1,1-diphenyl-2-picryl-hydrazine (DPPH) assays determines the antioxidant strength of the samples to reduce and scavenge radical compounds [55]. The antioxidant strengths majorly attributed to the total flavonoid and total phenolic content in the samples. Based on the results, the isolated *Lactobacillus* spp. from kefir demonstrated reduction activity in FRAP and scavenging activity in DPPH in accordance with TPC and TFC activities. These data are similar to a previous study done by Oh et al. [56], who reported that the antioxidant capacity measured by FRAP and DPPH assay are consistent with the enhancement of TPC and TFC during microbial fermentation. The report by Virtanen et al. [57] also emphasized that the antioxidant activity of the sample may differ based on the metabolic activity of different *Lactobacillus* species, and different strains within the same species. The summary of the results from isolation, characterization and antioxidant activities of *Lactobacillus* spp. from kefir samples in Malaysia are illustrated in Figure 9.

## 4. Materials and Methods

### 4.1. Kefir Grains

Ten samples of kefir grains were obtained from various areas in Selangor, Malaysia, and labelled A to J. All of these kefir grains were propagated in 10% *w*/*v* brown sugar solutions (CED, Selangor, Malaysia) [23]. The kefir water was fermented at room temperature for 24 h with daily transfer and was propagated under standardized conditions for at least three times to remove the influences resulting from different fermentation procedures of the supplier. The kefir grains were then be propagated until its biomass increased by 10% from its original weight.

### 4.2. Enumeration and Isolation of Lactic Acid Bacteria from Kefir Grains

The supernatant was discarded, and the kefir grains were strained and washed with mineral water (Cactus, Perak, Malaysia). Ten grams of kefir grains were suspended in 90 mL of sterile saline (0.85% *w*/*v*, pH 7.2–7.4) and homogenized with an electrical blender (Panasonic, Shah Alam, Malaysia) for 20 min. Serial decimal dilutions were prepared in the same diluent, and 0.1 mL was inoculated in triplicates by surface spreading on de Man, Rogosa and Sharpe agar (MRS) (Merck, Darmstadt, Germany). The dominant *Lactobacillus* spp. colonies that grew on MRS agar, which had the same colony appearances (in terms of shape, size, and color),were isolated on MRS (Merck, Darmstadt, Germany) and incubated at 37 °C under aerobic conditions for 48 h. After 48 h of incubation, the resulting colonies were enumerated, and the counts were expressed as the decimal logarithms of the colony-forming units per milliliter (log CFU/mL). Isolated colonies were cultivated in MRS broth (Merck, Darmstadt, Germany) at 37 °C for 48 h. For microbial genomic DNA extraction of the 10 samples, 1 mL of each homogenate was centrifuged for 2 min at 13,000× *g*. Total DNA from the pellets was extracted using a DNA extraction kit (Promega, Madison, USA) according to the manufacturer’s instructions. The DNA obtained was quantified using a Nanodrop apparatus (Implen, München, Germany).

### 4.3. Lactic Acid Bacteria Identification using 16S rRNA Sequence Analyses

Genomic DNA was isolated from all the bacterial isolates and used as template for PCR. Primers used for the amplification of part of 16S rRNA were (forward: 5′-GAGAGTTTGATCCTGG-3′; reverse: 5′-TACCGCGGCTGCTGGCAC-3′) and were selected based on a previous report [58]. The PCR mix (50 μL) contained 25 μL of Taq PCR Master Mix ((2.5 Units Taq DNA polymerase, 1 × PCR buffer, 1.5 mM MgCl_2_, and 200 μM of each deoxynucleotide triphosphate (dNTPs; QIAGEN, Crawley, UK)), 0.2 μM of each 16S rRNA universal primer, and 1 μg of DNA template. The contents of the tubes were mixed and placed in a SelectCycler IISBT9600 (Select BioProducts, Edison, New Jersey, USA) for an initial denaturation step at 94 °C for 3 min and for 30 cycles under the following conditions: 94 °C for 30 s, 60 °C for 30 s, and 72 °C for 2 min. A final cycle step at 72 °C for 10 min was performed before being cooled to 4 °C. The PCR product was checked by agarose gel electrophoresis, purified, and sequenced. The nucleotide sequences were used for sequence identity analysis through BLAST (Nucleotide BLAST, database 16S rRNA sequences) (http://www.ncbi.nlm.nih.gov/blast) [23,24]. The predominant *Lactobacillus* spp. that had the highest sequence identity matches with 16S rRNA gene were used for further analysis.

### 4.4. Determination Probiotics Properties of Isolated Lactobacillus spp. in the Gastrointestinal Tract Model

#### 4.4.1. Tolerance to Low pH Conditions

This assay was tested according to Leite et al. [59], with slight modifications. Briefly, 1 mL of overnight culture was harvested by centrifugation at 10,000 rpm for 5 min and cells were suspended in phosphate-buffered saline solution (PBS) at pH 6.5 to obtain an optical density of 0.5 at 600 nm (OD_600_). Cell suspensions were adjusted to pH 2.0, pH 3.0, and pH 4.0 with hydrochloric acid (HCl) (Systerm, Selangor, Malaysia) and incubated at 37 °C for 3 h. The pH tolerance of the cells was determined by enumerating the viable cells on MRS agar plates. The percentage of bacterial survival rate was calculated using the following equation:

(1) $$\mathrm{Survival}\;\mathrm{rate}\;(\%)\;=\;\frac{\mathrm{Final}\;\left(\mathrm{Log}\;\mathrm{CFU}/\mathrm{mL}\right)}{\mathrm{Initial}\;\left(\mathrm{Log}\;\mathrm{CFU}/\mathrm{mL}\right)\;}\times 100\%$$

#### 4.4.2. Bile Salt Tolerance Test

The bile tolerance assay was tested according to Leite et al. [59], with modifications. Overnight cultures of each *Lactobacillus* strain were harvested by centrifugation at 10,000 rpm for 5 min and cells were suspended in PBS at pH 6.5 to obtain an optical density of 0.5 at 600 nm (OD600). Cell suspensions were adjusted to 0.3% (*w*/*v*) oxgall and 0.5% (*w*/*v*) oxgall (Difco, Detroit, MI, USA), and incubated at 37 °C for 3 h. The bile tolerance was estimated by enumerating the viable cells on MRS agar plate and comparing viable cell counts in MRS with and without bile (ox gall). The percentage of bacterial survival rate was calculated using Equation (1).

#### 4.4.3. Adherence Assay

The ability of isolated *Lactobacillus* to adhere to the intestine was determined using HT-29 cells by using a method that was described by Kim et al. [60], with few modifications. Monolayer of HT-29 cells was prepared in a six-well tissue culture plate at 5 × 10^5^ cells/mL. The assay required HT-29 cells to achieve 90–100% confluence. Prior to the assay, the monolayer was washed thrice with PBS at pH 7.2–7.4. The overnight cultures of *Lactobacillus* in MRS broth were centrifuged for 10 min at 10,000 rpm and the pellets were re-suspended in antibiotic-free medium. Bacteria cells at 10^10^ CFU/mL (OD_600_) were added to the monolayer in six-well tissue culture plate in the presence of RPMI-1640 media without any antibiotic–antimycotic solution. The plate was then incubated for 2 h in 5% CO_2_ at 37 °C. The monolayer was washed thrice with PBS and fixed in methanol (Fisher Scientific, Fairlawn, NJ, USA) for 15 min. After fixing, the monolayer was washed thrice with PBS (pH 7.2–7.4), allowed to air-dry, and stained with Giemsa (Bio-Rad, Munich, Germany). The six-well plate was then examined microscopically under bright field microscope (Nikon Eclipse TS100, Tokyo, Japan). The adhesion ability was assessed in terms of viable colony counts. The number of viable cells of the *Lactobacillus* strains was counted by using the spread plate method on MRS agar. The percentage of bacterial adhesion to HT-29 cells was calculated as followed:

(2) $$\mathrm{Adhesion}\;\left(\%\right)=\frac{Adhered\;bacteria\;\left(CFU/mL\right)}{Total\;added\;bacteria\;\left(CFU/mL\right)}\times \;100\%.$$

#### 4.4.4. Scanning Electron Microscopy (SEM) of Adhered Isolated *Lactobacillus* toward Intestinal Cells

The ability of *Lactobacillus* to adhere to the intestine was determined using HT-29 cells by using a method described by Kim et al. [61], with few modifications. The HT-29 cell line was maintained in Roswell Park Memorial Institute 1640 (RPMI-1640) medium supplemented with 10% fetal bovine serum and 1% penicillin–streptomycin (Life Technologies, Carlsbad, CA, USA). Monolayer of HT-29 cells was prepared in a six-well tissue culture plate at a 5 × 10^5^ cells/well. The assay required HT-29 cells to achieve 90–100% confluency. Prior to the assay, the monolayer was washed thrice with PBS) (pH 7.2–7.4). The overnight culture of *Lactobacillus* in MRS broth was centrifuged for 10 min at 10,000 rpm and the pellets were re-suspended in an antibiotic-free medium. Bacteria cells at 10^10^ CFU/mL (OD_600_) were added to the monolayer in six-well tissue culture plate in the presence of RPMI-1640 media (Sigma-Aldrich, St. Louis, MO, USA) without any antibiotic–antimycotic solution. The plate was then incubated for 2 h in 5% CO_2_ at 37 °C. Cell monolayers were washed gently three times with 0.1 M phosphate buffer (pH 7.2) to remove any unbound bacteria. The cells were fixed with 2.5% (*v*/*v*) glutaraldehyde (Sigma-Aldrich St. Louis, USA) in 0.1 M phosphate buffer for 2 h at room temperature. Then, the cells were dehydrated in a graded ethanol series (50%, 70%, 80%, 90%, and 95% *v*/*v*) for 15 min each session (Systerm, Selangor, Malaysia), followed by two times dehydration step in 100% ethanol (Fisher Scientific, Fairlawn, NJ, USA) for 30 min. The cover slips containing the fixed cells were air dried at room temperature for 30 min, mounted on stubs, and coated with gold for 15 s. The specimens were then examined through SEM (FESEM; Hitachi, Japan).

#### 4.4.5. Antibiotic Susceptibility Test

This test was done according to Rajoka et al. [61], with few modifications. The activated cultures were swabbed on de Man Rogosa Sharpe (MRS) agar (Merck, Darmstadt, Germany) and incubated at 37 °C for 24 h. Isolated *Lactobacillus* spp. from kefir grains were tested for susceptibility toward 5 antimicrobial agents (Oxoid, Hampshire, UK): Vancomycin (30 μg), ampicillin (10 μg), penicillin G (10 IU)), tetracycline (30 μg), and gentamycin (10 μg), by Kirby–Bauer disc diffusion method. After 24 h, the zones of inhibition were measured for each sample. All samples were tested in triplicates.

#### 4.4.6. Hemolytic Test

Hemolytic assay was done according to Leite et al. [59]. All *Lactobacillus* spp. were plated on triplicate blood agar plates, containing 5% (*w*/*v*) sheep blood (Fisher Scientific, Fairlawn, NJ, USA), and incubated for 48 h at 37 °C. Blood agar plates were examined for signs of β-hemolysis (clear zones around colonies), α-hemolysis (green-hued zones around colonies), or γ-hemolysis (no zones around colonies).

### 4.5. Determination of Antioxidant Activity

#### 4.5.1. Total Phenolic Content (TPC) Assay

Total phenolic contents of isolated *Lactobacillus* spp. from kefir samples were determined by the Folin–Ciocalteu assay and the results were expressed in milligrams of gallic acid [62]. Briefly, supernatant of the isolated *Lactobacillus* spp. from kefir samples were incubated with Folin–Ciocalteu reagent (Sigma-Aldrich, St. Louis, USA) for 3–8 min. The mixture was then added with 0.08 mL of 7.5% sodium carbonate anhydrous (Sigma-Aldrich, St. Louis, USA) solution and incubated at room temperature for 2 h. The absorbance was measured at 765 nm using ELISA Plate Reader (Bio-Tek Instruments, Winooski, VT, USA).

#### 4.5.2. Total Flavonoid Content (TFC) Assay

Total flavonoid content of supernatant of isolated *Lactobacillus* spp. from kefir samples was determined by the aluminum chloride colorimetric method [62]. In brief, 0.15 mL supernatant of isolated *Lactobacillus* spp. from kefir was mixed with 0.09 mL 5% NaNO_3_ solution. After 5 min of incubation, 0.09 mL of 10% AlCl_3_ and the mixture was allowed to stand for 6 min. Then, 0.06 mL of 1 N NaOH solution was added and the final volume of the mixture was brought to 0.15 mL with distilled water. The mixture was allowed to stand for 15 min and the absorbance was measured at 510 nm using ELISA Plate Reader (Bio-Tek Instruments, Winooski, VT, USA). The TFC was calculated from a calibration curve and the result was expressed as mg Catechin/mg protein.

#### 4.5.3. 2,2-Diphenyl-2-picrylhydrazyl Assay

The free-radical-scavenging activities of the isolated *Lactobacillus* spp. from kefir samples were measured by the DPPH assay with Trolox (Sigma-Aldrich, St. Louis, USA) as the standard. Briefly, 50 μL of supernatant of the isolated *Lactobacillus* spp. from kefir samples were added to 250 μL of DPPH working solution and incubated in the dark for 30 min. The absorbance was measured in an ELISA Plate Reader (Bio-Tek Instruments, Winooski, VT, USA).

#### 4.5.4. Ferric Reducing Antioxidant Power Assay

The ferric reducing antioxidant power (FRAP) assay was done according to Thaipong et al. [56]. The working solution was prepared by adding 4 mL of 2,4,6-tri(2-pyridyl)-triazine (TPTZ) (Sigma-Aldrich, St. Louis, USA) and 4 mL Iron(III) chloride hexahydrate (FeCl_3_·6H_2_O) (Friendemann Schmidt, Parkwood, WA, USA) to 40 mL acetate buffer. The solution was warmed at 37 °C in the dark before use. Then, 20 µL supernatant of the isolated *Lactobacillus* spp. from kefir was added to a 96-well plate, mixed with 150 µL FRAP working solution, and then incubated for 10 min. The absorbance was measured at 593 nm, using an ELISA plate reader (Bio-Tek Instruments, Winooski, VT, USA). The results were calculated from the standard FeSO_4_ calibration curve and expressed as mg/mL Fe^2+^.

### 4.6. Statistical Analyses

Data are expressed as mean ± standard error (SE) calculated over three independent experiments performed in triplicate. SPSS version 20 (SPSS Inc., Chicago, IL, USA) was used to perform all statistical analysis. The statistical comparison analysis was done using one-way ANOVA, followed by Tukey’s post hoc test. Statistically significant data were considered when *p* < 0.05.

## 5. Conclusions

Overall, *L. harbinensis, L. plantarum*, and *L. paracasei* were the predominant groups of *Lactobacillus* spp. in 10 kefir samples in Malaysia. Isolated *Lactobacillus* spp. from kefir samples in Malaysia showed great probiotics potential. This was proved through probiotic screening assays where it had the ability to survive at low pH, tolerance to bile salt, and ability to adhere to HT-29 cells. Further study to investigate the bioactivity of this probiotic bacteria using in vivo system is necessary and important to understand the mechanism of probiotic actions and to further confirm the beneficial effect of this probiotic bacteria in daily consumption.

## Figures and Tables

**Table 1:** *Lactobacillus* spp. isolated from Malaysia’s kefir samples. Identified matches *Lactobacillus* spp. isolates by 16S rRNA gene sequencing analysis from GenBank database.

16S rRNA Sequences from Isolates	Accession No.	Matches to 16S rRNA Sequences from GenBank Database	Identity (%) with GenBank Database
A	KC415613.1	*L. harbinensis* strain B22	99%
	KX279364.1	*L. harbinensis* strain HBUAS5305	99%
	MH393129.1	*L. harbinensis* strain NBRC 100982	99%
B	KC415613.1	*L. harbinensis* strain B22	99%
	AY974809.1	*L. brevis strain* HDRS2	99%
	KF418816.1	*L. harbinensis strain* FQ003	99%
C	KC415613.1	*L. harbinensis* strain B22	99%
	KX279364.1	*L. harbinensis* strain HBUAS5305	99%
	KX279985.1	*L. sp MS6*	98%
D	EU559596.1	*L. plantarum* Gt2	98%
	KY041688.1	*L. plantarum* ZDY36a	98%
	MH472974.1	*L. plantarum* HBUAS52249	98%
E	MH392958.1	*L. paracasei* strain HBUAS52231	98%
	MH472956.1	*L. paracasei* strain HBUAS53273	98%
	MF083138.1	*L. casei* strain YQ116	98%
F	KC415613.1	*L. harbinensis* strain B22	99%
	KX279985.1	*L. sp MS6*	99%
	AY974809.1	*L. brevis strain* HDRS2	99%
G	MH472956.1	*L. paracasei* strain HBUAS52231	98%
	MH392958.1	*L. paracasei* strain HBUAS52231	98%
	KU851192.1	*L. casei* strain H19.9	98%
H	MG551235.1	*L. plantarum* strain NWAFU1558	98%
	KJ736728.1	*L. plantarum* strain Akhavan-Q3	98%
	EU637397.1	*L. plantarum* strain Y-2-9	97%
I	MH392958.1	*L. paracasei* strain HBUAS52231	99%
	MH472956.1	*L. paracasei* strain HBUAS53273	99%
	MF083138.1	*L. casei* strain YQ116	98%
J	MH620395.1	*L. plantarum* strain MSD1-4	98%
	CP0222294.1	*L. plantarum* strain DSR M2	98%
	CP028977.1	*L. plantarum* strain LQ80	98%

**Table 2:** Antibiotic susceptibility results against 10 isolated *Lactobacillus* spp. from Malaysia’s kefir samples.

Isolate	Diameter Zone Inhibition (mm)				
	VA	CN	AMP	TE	P
A	-	-	+++	+++	+++
B	-	-	+++	+++	+++
C	-	-	+++	+++	+++
D	-	-	+++	+++	+++
E	-	-	+++	+++	+++
F	-	-	+++	+++	+++
G	-	-	+++	+++	+++
H	-	-	+++	+++	+++
I	-	-	+++	+++	+++
J	-	-	+++	+++	+++

Note: Values indicate mean of triplicates. Resistant (-), moderately susceptible (+; inhibition zone: 10–20 mm), susceptible (++; inhibition zone: 21–30 mm), and very susceptible (+++; inhibition zone > 31 mm).

**Table 3:** Antioxidant activity of isolated *Lactobacillus* spp. from Malaysia’s kefir sample.

Isolate	TPC (mg GAE/mg protein)	TFC (mg catechin/mg protein)	FRAP (mM FRAP/μg protein)	DPPH (%)
A	21.94 ± 4.52 ^a^	9.12 ± 0.46 ^a,b^	0.25 ± 0.01 ^a^	63.10 ± 0.20 ^e^
B	58.26 ± 4.42 ^c,d^	10.39 ± 0.28 ^b,c^	0.97 ± 0.01 ^c^	54.33 ± 0.36 ^b^
C	45.41 ± 1.36 ^b,c^	10.76 ± 0.99 ^b,c^	1.46 ± 0.14 ^d^	60.21 ± 0.11 ^d^
D	39.71 ± 3.10 ^b^	7.51 ± 0.35 ^a^	0.64 ± 0.14 ^b^	48.43 ± 0.73 ^a^
E	60.42 ± 2.69 ^d^	12.61 ± 0.46 ^c^	1.25 ± 0.01 ^d^	53.67 ± 0.20 ^b^
F	75.14 ± 8.58 ^e^	19.13 ± 0.04 ^d^	1.76 ± 0.10 ^e^	67.40 ± 0.81 ^f^
G	115.97 ± 7.22 ^g,^*	58.94 ± 2.06 ^f,^*	2.81 ± 0.07 ^f^	76.79 ± 0.47 ^g,^*
H	99.20 ± 4.46 ^f^	36.29 ± 0.82 ^e^	2.58 ± 0.08 ^f^	58.70 ± 0.63 ^c^
I	50.93 ± 4.65 ^b,c,d^	12.24 ± 0.43 ^c^	1.81 ± 0.02 ^e^	48.91 ± 0.24 ^a^
J	39.93 ± 1.73 ^b^	7.72 ± 0.37 ^a^	0.79 ± 0.005 ^b,c^	49.50 ± 0.19 ^a^

Note: Comparison of total phenolic content, total flavonoid content, FRAP activity, and DPPH antioxidant activities of isolated *Lactobacillus* spp. from Malaysia’s kefir sample. ^a,b,c,d,e,f,g^ within the same column where the different superscript letters differ significantly (*p* < 0.05). * indicate mean values (*p* < 0.05).
